# Nanolabels Prepared by the Entrapment or Self-Assembly of Signaling Molecules for Colorimetric and Fluorescent Immunoassays

**DOI:** 10.3390/bios14120597

**Published:** 2024-12-06

**Authors:** Ning Xia, Yadi Li, Cancan He, Dehua Deng

**Affiliations:** Henan Province Key Laboratory of New Opto-Electronic Functional Materials, College of Chemistry and Chemical Engineering, Anyang Normal University, Anyang 455000, China; 19711087039@163.com (Y.L.); 13403862830@163.com (C.H.); ddh@aynu.edu.cn (D.D.)

**Keywords:** immunoassays, signaling molecules, nanocontainers, self-assembly, nanomaterials

## Abstract

Nanomaterials have attracted significant attention as signal reporters for immunoassays. They can directly generate detectable signals or release a large number of signaling elements for readout. Among various nanolabels, nanomaterials composed of multiple signaling molecules have shown great potential in immunoassays. Generally, signaling molecules can be entrapped in nanocontainers or self-assemble into nanostructures for signal amplification. In this review, we summarize the advances of signaling molecules-entrapped or assembled nanomaterials for colorimetric and fluorescence immunoassays. The nanocontainers cover liposomes, polymers, mesoporous silica, metal–organic frameworks (MOFs), various nanosheets, nanoflowers or nanocages, etc. Signaling molecules mainly refer to visible and/or fluorescent organic dyes. The design and application of immunoassays are emphasized from the perspective of nanocontainers, analytes, and analytical performances. In addition, the future challenges and research trends for the preparation of signaling molecules-entrapped or assembled nanolabels are briefly discussed.

## 1. Introduction

The quantitative detection of analytes shows an increasingly widespread application in different fields. Various analytical methods have been developed, including high-performance liquid chromatography and gas chromatography integrated with ultraviolet, fluorescence, Raman, or mass spectrometry. Despite the high detection performance, these methods always rely on expensive instruments, highly skilled personnel, and complicated operations. Thus, a series of novel detection techniques have been developed with specific recognition elements to capture the targets. At present, antibodies are still the most commonly used recognition elements in research and commercial bioaffinity assays despite many attempts to replace them with alternative receptors (e.g., aptamers, polymers, and artificial molecules). The specific antigen–antibody interaction with a high binding constant ensures the development of highly sensitive and selective immunosensing platforms [1]. For instance, immunoassay has been regarded as the gold standard for the sensitive detection of various biomarkers in clinical diagnosis. The targets are generally determined by using reporter-labeled antibodies or antigens to achieve signal generation and amplification in a competitive or sandwich format. In traditional immunosorbent assays, natural enzymes such as horseradish peroxidase and alkaline phosphatase are commonly employed to catalyze the conversion of substrate molecules into detectable products. However, natural enzymes have inherent drawbacks such as a high production cost, a short shelf life, sensitivity to environmental change, and low activity after binding, leading to an increasing demand for more effective alternatives [2,3].

With the rapid development of nanotechnology, nanomaterials have attracted great attention due to their excellent physical, chemical, optical, and electronic properties. Compared with traditional natural enzymes, nanomaterials have the advantages of simple preparation, low cost, high scalability, good stability, and signal multiplicity. Therefore, nanomaterials as the candidates for antibody or antigen-conjugated labels, named nanolabels, have shown broad prospects in developing novel immunosensing platforms [2]. After the immunoreactions on the sensor interface, the nanolabels can produce detectable signals directly or release a large number of signaling molecules for signal readout and amplification [2,3,4,5,6,7]. For example, various nanomaterials with enzyme-like catalytic activity defined as nanozymes have been exploited for bioassays since Fe_3_O_4_ nanoparticles were found to exhibit peroxidase catalytic activity, including noble metal nanoparticles, metallic oxides or sulfides, carbon-based nanostructures, and others [8,9,10,11]. Metal-containing nanoparticles and quantum dots attached to the target analytes can release a large number of metal ions via acid dissolution for electrical or optical assays [4]. Because each nanolabel entraps or is composed of tens or hundreds of signaling molecules or metal ions, the release-based strategy is more promising in the construction of highly sensitive, enzyme-free immunosensors.

Along with the widespread application of inorganic nanomaterials, many research efforts have also been focused on the development of organic molecule-based nanomaterials [12]. In addition to their low toxicity, the nanostructures composed of organic signaling molecules can be combined with other components, such as polymers or inorganic cores/matrices, to exhibit many useful properties, including the tunability of spectral characteristics, the potential for intrinsic responses to microenvironments, and possible multifunctionality. Such signaling molecules can be entrapped in nanocontainers or self-assemble into nanostructures to serve as signal labels for immunoassays. Over the last couple of years, a variety of materials such as liposomes, polymers, mesoporous silica, biological macromolecules, metal nanoparticles, metallic oxides, and carbon-based nanostructures have been explored as nanocontainers for drug delivery, bioimaging, theranostics, and sensors [13,14,15,16]. Furthermore, spontaneously organizing discrete components into highly ordered nanostructures facilitates the formation of nanomaterials with a desired size and multifunctionality [5,17,18]. Thus, the self-assembly of signaling molecules has attracted widespread attention in the field of bioassays. In consideration of the great potential of nanocontainers and self-assembled nanomaterials, some review papers have addressed their applications in different fields [5,19,20,21,22]. However, most of them only focus on one type of nanomaterial or component or the applications in fields other rather than immunoassays. In this work, we provide a comprehensive and systematic overview on the development of immunoassays with signaling molecules—entrapped or assembled nanomaterials—as signal labels. The design and application of such immunoassays are emphasized from the perspective of nanocontainers, analytes, and analytical performances. It is noticed that most of the entrapped or assembled signaling molecules are visible or fluorescent organic dyes, and colorimetric and fluorescence analysis have attracted growing attention in analytical applications owing to their unique virtues of simple operation, naked-eye signaling, and high sensitivity [23]. Thus, this review mainly specializes in the applications of signaling molecules—entrapped or assembled nanomaterials—in colorimetric and fluorescence immunoassays.

## 2. Signaling Molecules—Entrapped Nanomaterials

The hollow interior and large surface areas of nanocontainers improve the possibility of signal enhancement in bioassays. Their cavities or pores allow for the entrapment of a huge amount of signaling molecules and the attachment of multiple biological recognition elements. For analytical applications, the entrapped signaling molecules can be released by external stimuli, and alternately, the signal can be directly measured without the release of entrapped molecules. With the advancements in nanotechnology, various nanocontainers with large surface-to-volume ratios have been developed. In this section, the advances in the development of immunoassays with signaling molecules—entrapped nanomaterials—as signal labels are discussed based on different types of nanocontainers, and their analytical performances are shown in Table 1, including liposomes, polymers, mesoporous silica, metal–organic frameworks (MOFs), and metal, carbon, and protein-based nanosheets, nanoflowers or nanocages.

### 2.1. Liposomes and Polymers

**Figure 1:**
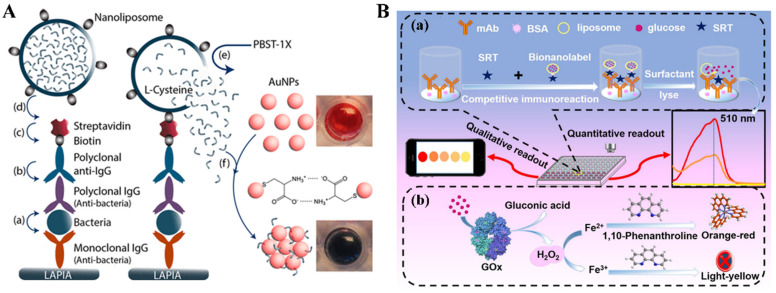
(**A**) Schematic of the liposome-amplified plasmonic immunoassay (LAPIA). The detection steps include the capture (**a**) and recognition (**b**) of the target, attachment of streptavidin (**c**), coupling of biotin-conjugated cysteine-contained liposomes (**d**), breakdown of the liposomes (**e**), and release of cysteine to trigger the aggregation of AuNPs (**f**) [27]. Copyright 2015 American Chemical Society. (**B**) Schematic illustration of signal-on competitive-type colorimetric immunoassay for the detection of streptomycin (STR) on monoclonal anti-STR antibody-coated microplate using glucose-loaded liposome as the signal tracer labeled with STR-bovine serum albumin (BSA) conjugate: (**a**) competitive-type immunoreaction and (**b**) glucose oxidase (GOx)-triggered the change of the Fe(II)-Phen system in the absorbance and visual color by the reaction of the produced H_2_O_2_ with iron(II) [41]. Copyright 2018 Elsevier.

Liposomes are artificial microscopic vesicles composed of a bilayer of hydrophobic lipids and polar heads oriented toward the extracellular solutions and inner cavities. Water-soluble and water-insoluble reagents can be encapsulated in the cavities or incorporated into the bilayer membrane of liposomes. They have been widely used as versatile carriers for therapeutic applications due to their low toxicity, easy biodegradability, and good biocompatibility [19,24,25]. Due to their high capacity for entrapping signal tags in the cavities, biofriendly liposomes have been widely used as carriers in the design of enzyme-free immunoassays. Small molecules, metal ions, and nanoparticles entrapped in liposomes can be released after the hydrolysis of liposomes induced by hydrolytic agents or surfactants such as Triton X-100 and Tween-20 [26,27,28,29,30,31]. The released species can provide visible/fluorescence signals directly or trigger additional chromogenic reactions. For example, visible dye methyl blue and fluorescence dyes sulforhodamine B, calcein as well as 5(6)-carboxyfluorescein have been entrapped inside liposomes for the design of colorimetric/fluorescence immunoassays of pathogens, bacteria, and proteins [32,33,34,35,36,37,38]. Bui et al. reported AuNPs-based colorimetric plasmonic immunoassays of pathogen and IgG with cysteine-loaded liposomes as signal labels [27]. This method is based on the cysteine-triggered aggregation of AuNPs based on the gold–thiol interactions and the formation of intermolecular hydrogen bonds between the free amine and carboxyl groups in cysteine. The schematic principle of the plasmonic immunoassay was depicted in Figure 1A and the detailed detection procedures were included in the figure caption. This method has been successfully used to determine the live pathogens of *Salmonella*, *Listeria*, and *Escherichia coli* (*E. coli*) in water and food samples. The detectable concentration for IgG is 6.7 attomolar, which is six orders of magnitude lower than the conventional ELISA. With a similar detection principle, Wang et al. reported a magnetic bead-based colorimetric plasmonic immunoassay of cystatin C for monitoring acute kidney injury with arginine-loaded liposomes as the signal labels [39]. After magnetic separation of the formed sandwich immunocomplexes, arginine-loaded liposomes were damaged by surfactant Triton ×100, and the positively charged arginine induced the aggregation of AuNPs via electrostatic and hydrogen bonding interactions. In addition, the plasmonic strategy has also been used to develop a signal-enhanced lateral flow immunosensor for the detection of *E. coli* O157:H7 by using biotinylated liposomes for entrapping branched polyethylenimine (BPEI) to trigger the aggregation of AuNPs [40].

Glucose can be readily measured by a glucometer, one of the most commonly used diagnostic devices. Glucose-loaded mesoporous nanocontainers have been exploited for the development of sensing strategies, and the signal could be monitored by glucometer or glucose-induced chromogenic reactions [41,42,43,44]. With glucose-encapsulated nanoliposomes as signal labels, colorimetric plasmonic immunoassays have been designed based on enzyme or nanozyme-triggered visual reactions [41,43]. For example, Ren et al. demonstrated that H_2_O_2_ produced from the glucose oxidase (GOx)-catalytic oxidation of glucose could oxidize Fe(II) into Fe(III), thus limiting the formation of orange-red Fe(II)-phenanthroline complexes (Figure 1B) [41]. Based on the change in solution color and absorbance intensity of Fe(II)-phenanthroline, the method was used for signal-on colorimetric immunoassay of streptomycin (STR) in a competitive format.

**Figure 2:**
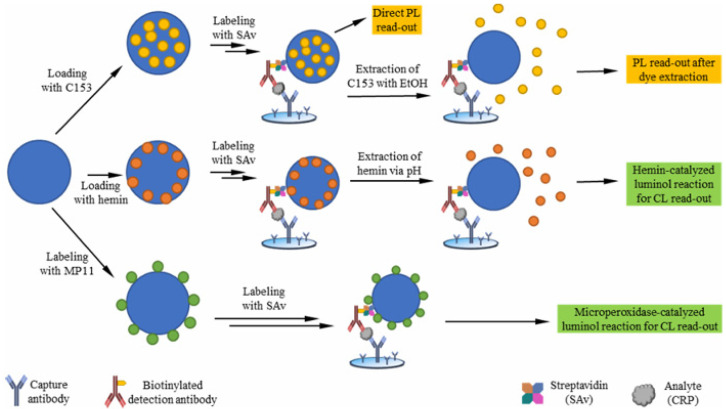
Schematic presentation of the heterogeneous sandwich immunoassay with PSP for loading load C153, hemin, or microperoxidase MP11 based on different signal generation strategies and photo/chemiluminescence detection [51]. Copyright 2024 American Chemical Society.

Polymers in the formation of spherical or granular nanoparticles are excellent candidates as carriers for the loading of signaling molecules due to their good conductivity, biocompatibility, and functionality [45,46,47,48]. Swelling is a straightforward method for loading hydrophobic molecules into commercially available stabilized polymer colloids [49,50]. Recently, Geißler et al. proposed heterogeneous sandwich immunoassays using carboxy-functionalized polystyrene particles (PSPs) as carriers for loading dye coumarin 153 (C153) or catalyzer hemin via the swelling method and to immobilize hemin-based microperoxidase MP11 via a covalent coupling reaction (Figure 2) [51]. C153 was released from PSP by ethanol to produce a fluorescence signal, and hemin was released by a buffer solution (pH 11) to catalyze the luminol reaction enabling chemiluminescence detection. The analytic performance was comparable to that labeled with hemin-based microperoxidase MP11. In this work, PSP was labeled with streptavidin for the conjugation of biotinylated antibody. The MP11-labeled chemiluminescence immunoassay for C-reactive protein (CRP) detection exhibited a detection limit of 0.1 ng/mL, which was lower than that of ELISA (0.13 ng/mL).

### 2.2. Silica-Based Mesoporous Materials

**Figure 3:**
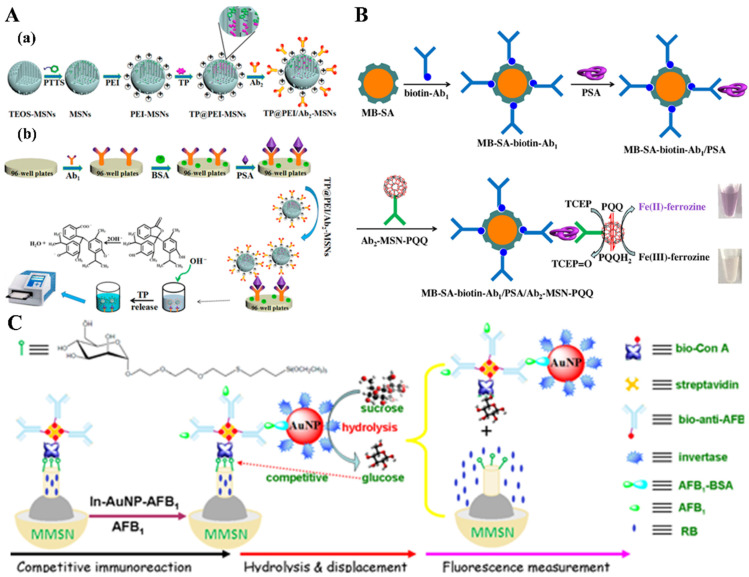
(**A**) (**a**) Synthesis and derivatization of TP@PEI/Ab_2_-MSNs and (**b**) steps of the enzyme-free immunosorbent assay of PSA using for amplified colorimetric detection in a 96-well plate [63]. Copyright 2018 American Chemical Society. (**B**) Schematic illustration of the magnetic bead (MB)-based colorimetric immunoassay of PSA by the redox cycling with Ab_2_-MSN-PQQ as the nanolabel [64]. Copyright 2019 Elsevier. (**C**) Schematic illustration of the fluorescence immunoassay based on target-induced competitive displacement reaction between glucose and mannose for Con A accompanying cargo (rhodamine B) release from magnetic mesoporous silica nanoparticles (MMSNs) [65]. Copyright 2013 American Chemical Society.

In contrast to liposomes and organic polymers, silica-based mesoporous materials show incomparable potential as the carriers for loading signaling molecules and recognition elements because of their excellent thermal and chemical stability [20,52,53,54,55]. Such mesoporous materials are endowed with the properties of inorganic materials and exhibit the advantages of large surface area, adjustable pore size, and adjustable hydrophobic or hydrophilic properties [56,57,58]. These unique characteristics pave the way for the loading of a large number of signal species. Usually, most of the silica-based nanomaterials are white or colorless and do not fade via treatment with harsh conditions such as light, heat, acid, and alkali. Thus, they are good candidates as optical labels for signal conversion and amplification after being dyed with organic dyes [59,60,61]. The colored silica-based nanomaterials show the inherent advantages of bright color, easy preparation, and good biocompatibility. Sun et al. reported the magnetic colorimetric immunoassay of *Salmonella pullorum* (*S. pullorum*) by using blue 21-loaded silica nanoparticles (blue-SiNPs) as the signal label [62]. In this work, blue-SiNPs were prepared by doping colorimetric indicator C.I. reactive blue 21 into SiNPs by a reverse microemulsion method, and the indicator was released from the nanoparticles using a 5 mM NaOH solution to produce an absorbance at 675 nm. Shao et al. reported the colorimetric immunoassay of prostate-specific antigen (PSA) using phenyltrimethyloxysilane-functionalized mesoporous silica nanoparticle (MSN) to entrap pH-indicator thymolphthalein (TP) using hydrophobic and π–π interactions (Figure 3A) [63]. To detect PSA, the TP-loaded MSN was coated with polyethylenimine (PEI) to favor the attachment of a negatively charged detection antibody. After the immunoreaction, the entrapped TP dyes were released from MSN by 0.1 M NaOH to produce a visual signal. Pyrroloquinoline quinone (PQQ) is a redox cofactor for many PQQ-dependent enzymes. We found that PQQ could promote the redox cycling between Fe(III)-ferrozine and tris(2-carboxyethyl)phosphine (TCEP), thus leading to the formation of dull red Fe(II)-ferrozine complexes (Figure 3B) [64]. Using MSN as the carrier to load PQQ, magnetic immunoassays were achieved for the determination of PSA with a detection limit down to 1 pg/mL.

Besides colorful dyes, fluorescence dyes have also been entrapped in silica-based mesoporous materials for immunoassays [65,66,67]. For example, Ghafary et al. developed a magnetic nanoparticle-based immunoassay of hepatitis B virus surface antigen (HBsAgs) using MSN to entrap fluorescence dye Rhodamine B (RB) [67], in which ethanol was used to release the entrapped RB molecules. Tang et al. designed an immunosensor for AFB_1_ detection using a target-induced displacement reaction to release fluorescence dyes entrapped in protein-gated magnetic MSN (MMSN) (Figure 3C) [65]. In this method, MMSN was loaded with rhodamine B (RB) and functionalized with mannose-terminated silane for the capture of concanavalin A (Con A) via the carbohydrate–protein interaction. Anti-AFB_1_ antibody was linked to the Con A-functionalized MMSN for the capture of gold nanoparticles (AuNPs) modified with invertase and BSA–AFB_1_ conjugate. Invertase on the surface of AuNPs hydrolyzed sucrose into glucose that could compete with mannose to bind Con A, thus resulting in the release of Con A from MMSN and the opening of a molecular gate due to the uncapping of MMSN. In this case, the entrapped RB dyes were released from the pores. In the presence of AFB1, the competitive immunoreaction between AFB1 and BSA–AFB_1_ prevented the capture of invertase and BSA–AFB_1_ conjugate-modified AuNPs, thus limiting the hydrolysis of sucrose into glucose and the release of entrapped RB.

Antibodies can be used as gatekeepers to regulate the release of indicators [68]. Martínez-Máñez’s group first reported an antibody-gated indicator delivery system using sulfathiazole as the antigen and external stimuli [69]. Briefly, the outer surface of MSN was modified with hapten and coupled with antibody specifically. The presence of antigen resulted in the uncapping of the pore and the liberation of the entrapped fluorescent dyes. Based on the specific interaction between antibody and antigen, this antibody-gated indicator delivery system exhibited high selectivity and sensitivity, especially in a complicated matrix. Furthermore, several groups integrated the system with lateral flow assays and optimized the immunochemical response with enhanced detection performance [70,71,72,73,74]. For example, they reported an MSN-based antibody-gated indicator delivery system for the detection of triacetone triperoxide (TATP) [75]. In this study, the hapten heterology and the type of both indicator dyes and host materials were investigated for the best performances in terms of sensitivity, selectivity, and assay time. Subsequently, a slightly mismatching hapten was used to functionalize the surface of MSN with high-affinity antibodies as “caps”. Under the optimized conditions, the response time was shortened from hours to less than 5 min.

### 2.3. MOFs

**Figure 4:**
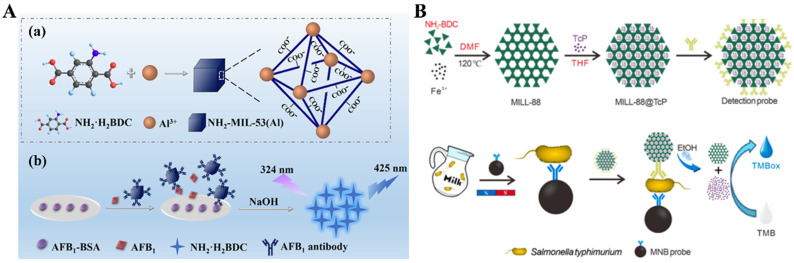
(**A**) (**a**) Preparation of MOFs NH_2_-MIL-53(Al) and (**b**) schematic illustration of competitive FIA of AFB1 [81]. Copyright 2019 American Chemical Society. (**B**) Schematic illustration of the synthetic procedure of MILL-88@TcP nanozyme-based detection probe (**top**) and the procedure of this developed N-ELISA for *S. typhimurium* detection in milk (**bottom**) [89]. Copyright 2024 Elsevier.

MOFs are a type of nanoporous hybrid material formed by the self-assembly of inorganic and organic building blocks. Under acidic or alkaline conditions, some metal ions in MOFs could be released to induce signal changes as a result of the formation of metal complexes or enzyme-like catalytic reactions [21,76,77]. Besides metal ions, some colorful or fluorescent ligands in MOFs could be used as signal indicators to develop biosensors [78,79,80]. For example, Wei et al. suggested that NH_2_-MIL-53(Al) MOFs can be directly used as the signal labels of fluorescent immunoassays due to the fluorescent emission of NH_2_-H_2_BDC ligands (Figure 4A) [81]. After treatment with an alkaline solution (10 mM NaOH), the antibody-modified NH_2_-MIL-53(Al) MOFs anchored on the microplate well were hydrolyzed to release a large amount of fluorescent NH_2_-H_2_BDC, achieving the detection of AFB1 in a competitive format.

The porosity of MOFs is usually higher than that of commercial nanoporous adsorbents, such as zeolites, silica gel, and activated carbon [21]. In addition, biorecognition elements such as antibody and DNA can be readily attached to the surface of MOFs by physical adsorption, covalent bonding or metal coordination interactions. Thus, MOFs serve as excellent carriers for different substances (e.g., metal nanoparticles, organic dyes, enzymes) [79,82,83,84]. Wang et al. reported the colorimetric immunoassay of PSA using the zeolitic imidazolate framework-8 (ZIF-8 MOF) as the nanocarrier to entrap the signaling molecule methyl yellow (MY) [85]. The ZIF-8@MY was prepared using the one-pot coprecipitation method and then modified with anti-PSA for the recognition of PSA. The hydrolysis of captured ZIF-8@MY using an acid solution led to the release of numerous MY probes, producing an obvious visual signal at 510 nm. Yan et al. developed a color-fluorescence dual-modal ELISA platform for the detection of CEA using ZIF-8 MOF as the carrier load TP indicators and carbon dots (CDs) [78], in which TP was released by 0.1 M NaOH.

Redox-active metal porphyrins have usually been used to mimic the activity of natural enzymes [86,87]. Iron porphyrin (TcP) as a molecular artificial enzyme exhibits HRP-mimicking catalytic activity of HRP [88]. Encapsulation of iron porphyrin in a suitable scaffold can favor the production of a core-shell structured nanozyme. Dual et al. suggested that TcP could be encapsulated within MILL-88 MOF in an inclusion structure, and its catalytic activity was well recovered under ethanol-induced release (Figure 4B) [89]. Due to the high catalytic activity of both MILL-88 and TcP, the colorimetric immunoassays showed a high sensitivity for the detection of *Salmonella typhimurium* (*S. typhimurium*) by using the MILL-88@TcP nanozyme as the signal labels. The detection limit is 500-fold lower than that of the traditional HRP-based ELISA.

**Figure 5:**
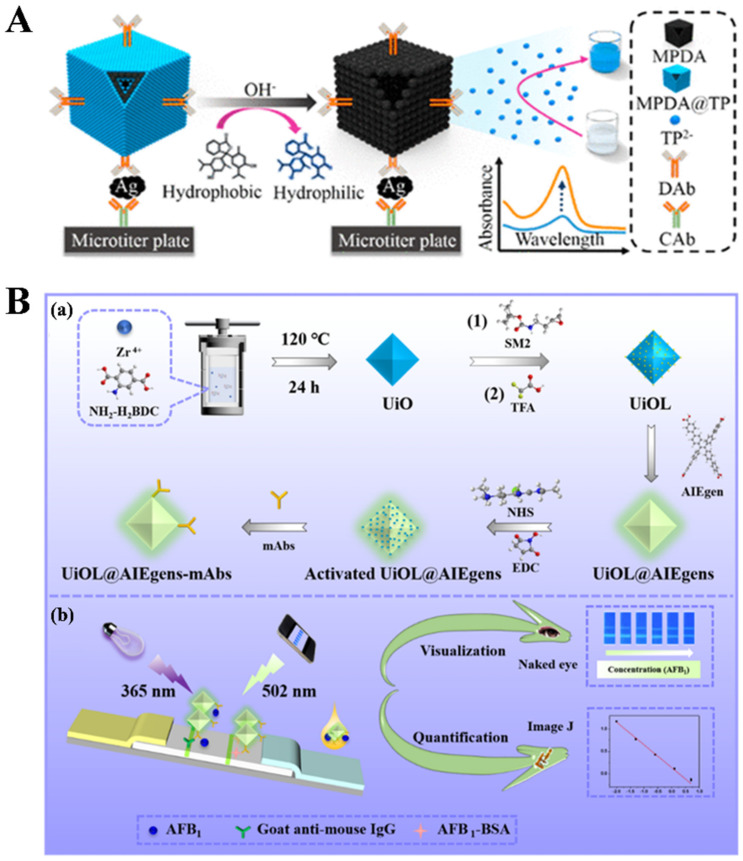
(**A**) Schematic representation of MPDA@TP-linked immunosorbent assay (MLISA) for α-fetoprotein (AFP) on anti-AFP capture antibody (CAb)-modified microplate using anti-AFP detection antibody (DAb)-labeled MPDA@TP with a sandwich-type immunoreaction mode [92]. Copyright 2018 American Chemical Society. (**B**) The synthesis of UiO, UiOL, UiOL@AIEgens, and UiOL@AIEgens-mAbs probe (**a**), and the UiOL@AIEgens-based POC LFIS for visual and quantitative dual-modal detection of AFB1 (**b**) [96]. Copyright 2024 Elsevier.

In addition, MOFs can be used as templates or precursors to prepare functional materials due to their fascinating structural topology and functional tunability. Such derived porous or hollow nanostructures have been utilized for gas adsorption, energy storage, bioassays, catalysis, and drug delivery [90]. Considering the rich functional groups, polydopamine (PDA) has also been used as the carrier of drugs and dyes [91]. To integrate the merits of PDA and MOFs, Ren et al. prepared hollow metal-polydopamine (MPDA) frameworks and used them as the carriers to load hydrophobic TP dyes via π-stacking interactions (Figure 5A) [92]. The resulting MPDA@TP was modified with an antibody as the signal label for the colorimetric immunoassay of AFP. After treatment with an alkaline solution, the entrapped TP molecules were released from the nanostructures in the format of hydrophilic TP^2−^ ions. This led to the change in solution color from colorless to deep blue and the increase in absorbance intensity at 595 nm.

Aggregation-induced emission luminogens (AIEgens) exhibit remarkable optical properties and strong resistance toward photobleaching [93,94,95]. Wu et al. established a signal-enhanced lateral flow immunosensor for AFB1 detection using zirconium UiO MOF to carry a linker-enriched AIEgen (Figure 5B) [96]. The UiO MOF was prepared with ZrCI_4_ as the metal ion cluster and 2-aminoterephthalic acid (NH_2_-H_2_BDC) as the organic ligand. The amino-protective tert-butyl (2-(oxiran-2-yl)ethyl)carbamate (SM2) was conjugated with NH_2_-H_2_BDC through a ring-opening reaction. The protective group was removed by trifluoroacetic acid (TFA) to produce amino-functionalized UiO (UiOL). Then, AIEgen was linked to the UiOL by an amidation reaction between the carboxyl group in AIEgen and the amino group on the UiOL surface. The UiOL@AIEgen with green fluorescence emission was further modified with anti-AFB1 monoclonal antibody (mAb) for the competitive immunoassay of AFB1. The competitive antigen–antibody reaction between AFB1 in the sample solution and AFB1-BSA immobilized on the T line limited the capture of UiOL@AIEgen, achieving the visual and smartphone-assisted dual-modal quantitative detection of the target.

### 2.4. Others

**Figure 6:**
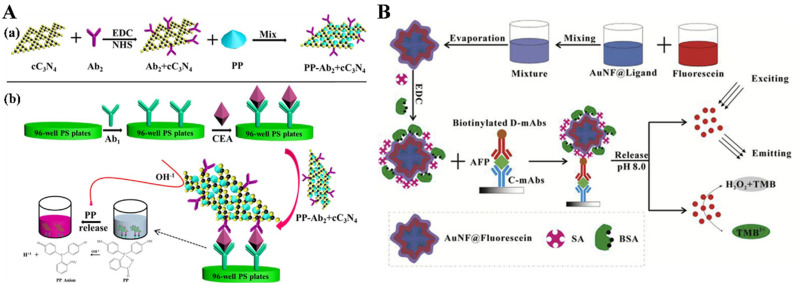
(**A**) (**a**) The preparation process for the signal label of PP-Ab_2_-cC_3_N_4_, and (**b**) schematic illustration of the PILISA for the detection of CEA in 96-well PS plates [100]. Copyright 2017 Elsevier. (**B**) Schematic of AuNF@Fluorescein@SA preparation, and AuNF@Fluorescein@SA-based dual-mode fluorescent and colorimetric immunoassay [101]. Copyright 2018 Elsevier.

Metal, carbon, and protein-based nanosheets, nanoflowers or nanocages with cubic or spherical hollow structures can be used for the entrapment of signaling molecules [15,97,98,99]. Shao et al. used carboxylic functionalized carbon nitride (cC_3_N_4_) nanosheets to load phenolphthalein (PP) dyes via a hydrophobic interaction and immobilize a second antibody (Ab_2_) via an amidation coupling reaction for immunoassays of protein biomarkers (Figure 6A) [100]. After the immunoreactions, the changes in color solution and signal intensity were monitored by the alkali solution-triggered release of PP molecules from cC_3_N_4_. Zhou et al. reported a fluorescent/colorimetric dual-mode immunosensing platform by using gold nanoflower (AuNF) to load fluorescein molecules for signal output. (AuNF@Fluorescein) (Figure 6B) [101]. AuNF was modified with a thiolated hydrophobic carboxyl ligand with good biocompatibility and solubility for fluorescein encapsulation and antibody immobilization. At a pH of 8.0, the entrapped fluorescein dyes were released from the hydrophobic wallet of AuNF, thus producing a strong fluorescent signal. Meanwhile, the released fluorescein could catalyze the oxidation of TMB using H_2_O_2_, thereby allowing for colorimetric immunoassay of AFP with a sensitivity 15-fold higher than that of the HRP-based method.

**Figure 7:**
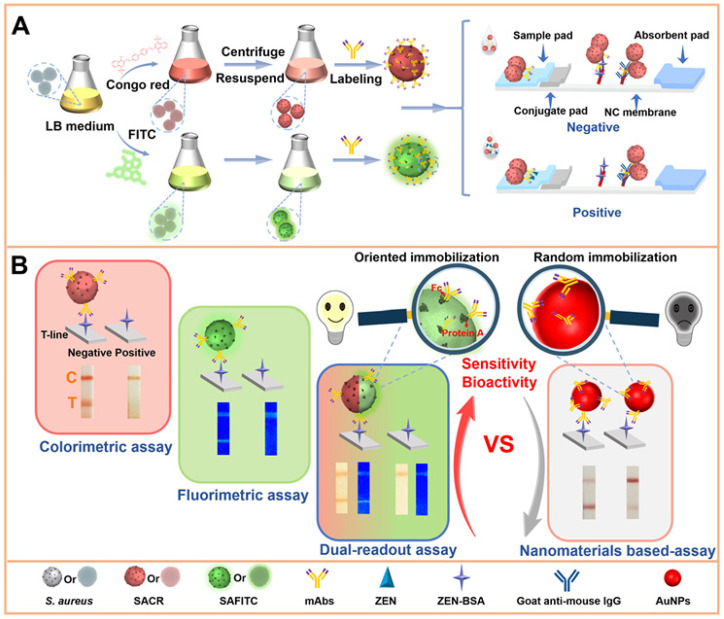
(**A**) Synthetic procedure for SAD carriers and their applications in ICAs. (**B**) Comparison of SAD-ICAs with three modes and a traditional nanomaterial—ICA (take AuNPs as an example)—for the detection of ZEN [102]. Copyright 2021 American Chemical Society.

Nanovesicles have been widely used for drug delivery due to their hollow structure, unique size, and intrinsic physicochemical properties. Pu et al. found that allochroic agents (phenolphthalein, methyl red, and bromothymol blue) can be entrapped in 3D gold nanovesicles (GNVs) as the indicators of colorimetric immunoassays of N-terminal pro B type natriuretic peptide (NT-proBNP), creatine kinase-MB (CK-MB), and cardiac muscle troponin T (cTnT) [97]. The entrapped dyes in GNVs were released by NaOH solution (pH 12) at 90 °C. Besides inorganic and organic materials, microorganisms have also been employed as carriers of load dyes for bioassays. Recently, Bu et al. developed immunochromatographic assays (ICAs) for point-of-care testing (POCT) by employing *Staphylococcus aureus* (*S. aureus*) to load the dyes of congo red (CR) and fluorescein isothiocyanate (FITC) (Figure 7) [102]. The signal labels of SACR, SAFITC, and SACR-SAFITC were successfully applied for the colorimetric, fluorescent or dual-modal assays of zearalenone (ZEN) in corn, peanut, millet, and bean samples. In this work, the dyes were incorporated into *S. aureus* by in-situ assembly, and anti-ZEN was directly targeted by *S. aureus* through the interaction between the Fc region of the antibody and the protein A expressed on the surface of *S. aureus*. The method showed several conspicuous advantages due to the large surface-to-volume ratio of *S. aureus*, the simple procedure for dye loading, and the site-directed labeling of the antibody on the carrier.

**Table 1:** Signaling molecules—entrapped nanomaterials—as signal labels.

Signal Label	Target	Linear Range	Detection Limit	Ref.
Liposome@fluorescein	PA	4.1–5000 ng/mL	4.1 ng/mL	[28]
Liposome@fluorescein	IgG	0.1–100 fg/mL	2 pg/mL	[32]
Liposome@SRB	ACAs	1.1–19.9 GPL units	2 GPL units	[34]
Liposome@SRB	CT	0.1–10 fg/mL	0.06 fg/mL	[35]
Liposomes@arginine	Cys C	10–100 ng/mL	4.32 ng/mL	[39]
Liposomes@BPEI	*E. coli*	300–600 CFU/mL	100 CFU/mL	[40]
Liposome@glucose	STR	0.001–20 ng/mL	0.4 pg/mL	[41]
Liposome@glucose	AFB1	0.001–10 ng/mL	0.6 pg/mL	[42]
Liposome@glucose	PSA	0.1–10^11^ pg/mL	53 fg/mL	[43]
PSP@C153	CRP	12–45 ng/mL	4.9 ng/mL	[51]
SiNPs@blue	*B. A*	1.5 × 10^3^–1.5 × 10^8^ CFU/mL	450 CFU/mL	[60]
SiNPs@blue	*S. pullorum*	4.4 × 10^2^–4.4 × 10^7^ CFU/mL	44 CFU/mL	[62]
MSN@TP	PSA	0.5–8000 pg/mL	0.36 pg/mL	[63]
MSN@PQQ	PSA	5–500 pg/mL	1 pg/mL	[64]
SiNSs@fluorescein	*E. coli*	4–4 × 10^8^ CFU/mL	3 CFU/mL	[66]
MMSN@RB	AFB1	0.01–5 ng/mL	8 pg/mL	[65]
ZIF-8@TP/CDs	CEA	10–500 pg/mL	10 pg/mL	[78]
NH_2_-MIL-53	AFB1	0.05–25 ng/mL	30 pg/mL	[81]
ZIF-8@MY	PSA	0.001–1 pg/mL	0.67 pg/mL	[85]
MILL-88@TcP	*S. typhimurium*	1.2 × 10^3^–1.2 × 10^6^ CFU/mL	168 CFU/mL	[89]
MPDA@TP	AFP	10–1000 pg/mL	2.3 pg/mL	[92]
UiOL@AIEgens	AFB1	0.01–5 ng/mL	3 pg/mL	[96]
GNVs@PP, GNVs@MR, GNVs@BB	CK-MB, cTnT, NT-proBNP,	0.1–2 × 10^5^ ng/mL, 1–2 × 10^3^ ng/mL, 0.1–5 × 10^3^ pg/mL	70 pg/mL, 910 pg/mL, and 7.8 pg/mL	[97]
cC_3_N_4_@PP	CEA	0.5–100 ng/mL	0.34 pg/mL	[100]
AuNF@FL	AFP	0.01–10 pg/mL	29 fg/mL	[101]
SACR	ZEN	0.02–12 ng/mL	45 pg/mL	[102]

Abbreviation: PA, protective antigen; SRB, sulforhodamine B; ACAs, anticardiolipin antibodies; CT, cholera toxin; Arg, arginine; Cys C, Cystatin C; BPEI, branched polyethylenimine; *E. coli*, *Escherichia coli O157:H7*; STR, streptomycin; AFB1, aflatoxin B1; PSA, prostate specific antigen; PSP@C153, coumarin153-stained polystyrene particles; CRP, C-reactive protein; SiNPs, hollow silica nanospheres; *B. A*, *Brucella Abortus*; *S. P*, *Salmonella pullorum*; MSN, mesoporous silica nanoparticle; PQQ, pyrroloquinoline quinone; TP, thymolphthalein; MMSN, magnetic mesoporous silica nanoparticles; ZIF-8, zeolitic imidazolate framework-8; CDs, carbon dots; CEA, carcinoembryonic antigen; NH_2_-MIL-53, Al^3+^-based metal–organic framework; MY, methyl yellow; TcP, iron porphyrins; *S. typhimurium*, *Salmonella typhimurium*; MPDA, metal-polydopamine framework; UiOL@AIEgens, zirconium-based MOF carrying a linker-enriched aggregation-induced emission luminogens; GNVs, 3D gold nanovesicles; PP, phenolphthalein; MR, methyl red; BB, bromothymol blue; CK-MB, creatine kinase-MB; cTnT, cardiac muscle troponin T; NT-proBNP, N-terminal pro B type natriuretic peptide; AFP, alpha-fetoprotein; AuNF, gold nanoflower; SACR, Congo red-embedded *Staphylococcus aureus*; ZEN, zearalenone.

## 3. Signaling Molecules—Assembled Nanomaterials

**Figure 8:**
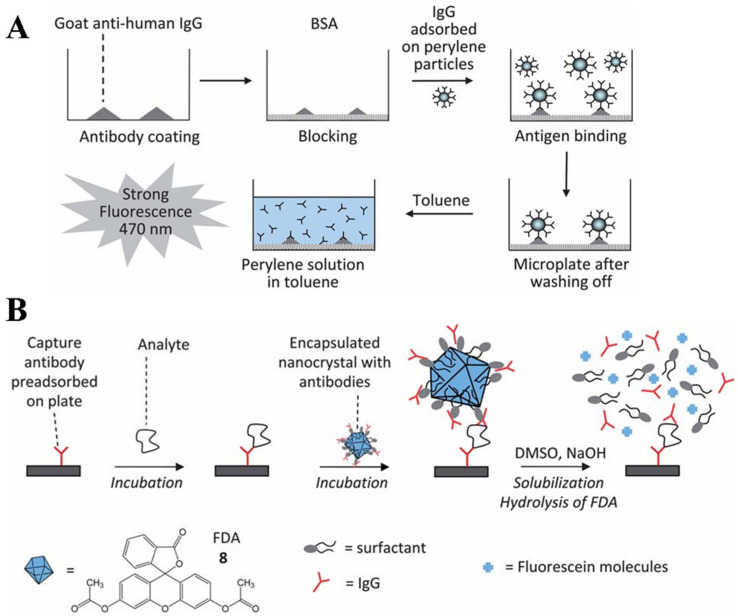
(**A**) Principle of immunoassay using antigen-decorated perylene microparticles [109]. Copyright 2000 Elsevier. (**B**) Principle of a sandwich fluorescent immunoassay using nanocrystalline fluorescein diacetate (FDA) conjugates [103]. Copyright 2004 American Chemical Society.

**Figure 9:**
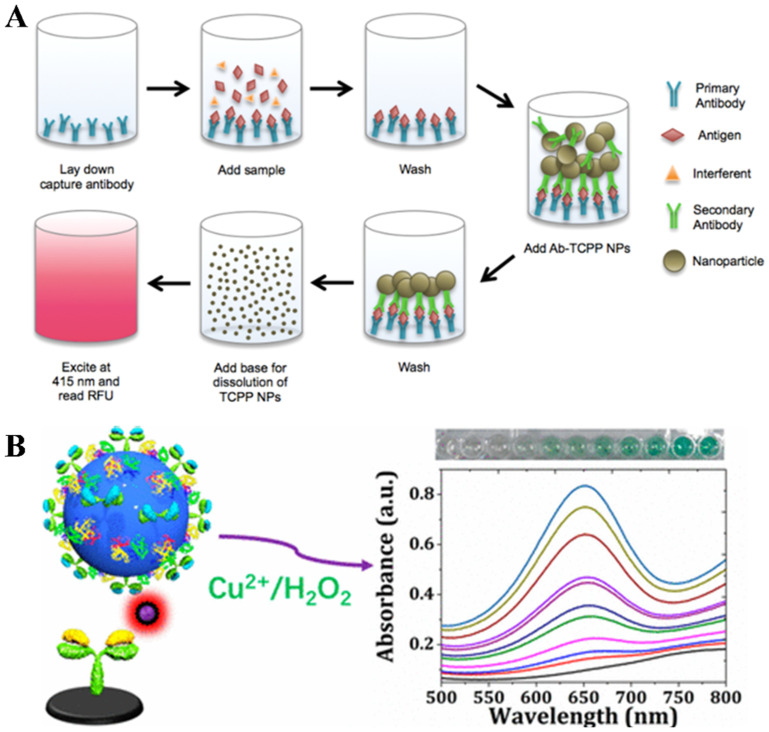
(**A**) Workflow of porphyrin nanoparticle-based signal amplification sandwich assays for the detection of biomolecules [112]. Copyright 2016 American Chemical Society. (**B**) Scheme of sandwich-type TLISA for the detection of IL-6 [115]. Copyright 2019 American Chemical Society.

Self-assembly is a process of spontaneously organizing distributed components into highly ordered structures, facilitating the formation of molecular superstructures with desired size and multifunctionality [17]. The strategy is highly attractive for manufacturing nanocomposites due to its uniformity, versatility, and ease of design. For bioassays, the self-assembly of individual disordered signaling probes into nanostructures can improve the detection sensitivity. Additionally, multifunctional probes with recognition capacity and catalytic properties can be prepared, and a large number of signaling molecules can be accumulated to enhance the signal intensity. Generally, the self-assembly of small molecules is mainly driven by non-covalent interactions such as electrostatic, hydrophobic, hydrogen bonding and van der Waals forces. For example, hydrophobic organic dyes are prone to aggregation-induced quenching (ACQ) due to reabsorption, energy transfer, and other processes. Organic dye-based nanomaterials have been used in aqueous environments for imaging and bioassays [5]. They can be stabilized by surfactants or polymers to prevent further aggregation and provide rich groups for the modification of biorecognition elements. In principle, organic dye-based nanomaterials can be prepared by a “top-down” or “bottom-up” method. For example, the grinding of fluorescein diacetate (FDA) in aqueous surfactant media can lead to the formation of nanocrystals with an average of 107 nm [103]. Compared to the “top-down” approach, the “bottom-up” strategy based on the self-assembly of organic dyes via weak non-covalent interactions is the most frequently used. One of the most popular techniques is the solvent exchange method, especially the reprecipitation method developed by Nakanishi’s group [104]. Such methods usually involve the preparation of a concentrated solution of dyes in hydrophilic organic solvents and the rapid injection of a few drops of the solution into a water or “bad solvent” aqueous solution under vigorous stirring. Due to the sudden change in solubility, the hydrophobic dyes may crystallize to form small nanocrystals, and their sizes are dependent on experimental conditions (e.g., solution temperature, dye concentration, stabilizer property, aging time, and solvent polarity). With organic dye-based nanocrystals as signal labels, fluorescent immunoassays were widely developed in the early 2000s (Table 2) [103,105,106,107,108,109,110,111]. For example, Kamyshny et al. used perylene-assembled microparticles to adsorb antigens for a competitive immunoassay of IgG (Figure 8A) [109]. The perylene microparticles captured on the plate well were dissolved by toluene into monomers with strong fluorescence emission. This method showed high sensitivity due to the extremely high dye-to-protein molar ratio, but it required the use of a large number of biorecognition elements during the modification of perylene microparticles via the reprecipitation method, increasing the detection cost. To resolve this problem, various strategies have been proposed to prepare and modify FDA nanocrystals for fluorescence immunoassays [103,105,107,110]. Typically, Renneberg’s group demonstrated that FDA nanocrystals could be encapsulated in a shell of PEG-amine-modified phospholipids, providing a surface for antibody attachment (Figure 8B) [103]. The captured nanocrystals were dissolved by the mixture of DMSO and NaOH, releasing abundant FDA dyes for signal amplification. In addition, Gibson et al. suggested that tetra(4-carboxyphenyl)porphyrin (TCPP) monomers could be easily assembled into nanostructures serving as the signal labels of fluorescent immunoassays of rabbit IgG and plasmodium falciparum histidine-rich protein II (pfHRPII) (Figure 9A) [112]. Antibodies were conjugated onto the surface of TCPP nanoparticles (TCPP NPs) with a sulfo-NHS-LC-diazirine photoreactive linker. After the immunoreactions, the captured TCPP NPs were dissolved by 0.1 M NaOH, releasing individual TCPP molecules that could be determined by monitoring the fluorescence intensity at 648 nm with an excitation wavelength of 415 nm.

In addition to hydrophobic fluorescent dyes, some small drug molecules can self-assemble into carrier-free nanodrugs using the reprecipitation method. Inspired by this fact, self-carried nanolabels by the self-assembly of small signaling molecules were prepared and used for immunoassays [113,114,115]. Typically, Jiao et al. found that hydrophobic 3,3′,5,5′-tetramethylbenzidine (TMB) molecules can rapidly assemble into NPs due to the sudden change in the solvent environment (Figure 9B) [115]. In the “one-pot” reprecipitation process, the recognition element of antibody and the stabilizing agent of bovine serum albumin (BSA) were accommodated with TMB NPs via hydrogen-bonding and van der Waals interactions. The all-inclusive signal labels were used for colorimetric immunoassay of inflammatory biomarker interleukin-6 (IL 6). In this method, the captured TMB NPs were dissolved in ethanol and the released TMB molecules were oxidized by H_2_O_2_ with Cu^2+^ as the catalyst. After that, pH-responsive allochroic NPs were prepared by the self-assembly of hydrophobic organic dyes—TP, PP and curcumin (CUR)—and used for visible assays of pathogenic bacteria—*Escherichia coli* (*E. coli*) and *S. typhimurium*—and protein biomarkers—estrogen receptor (ER), progesterone receptor (PR), and human epidermal growth factor receptor-2 (HER2) [116,117]. In this method, the allochroic NPs were dissolved in NaOH solution (pH 13).

**Figure 10:**
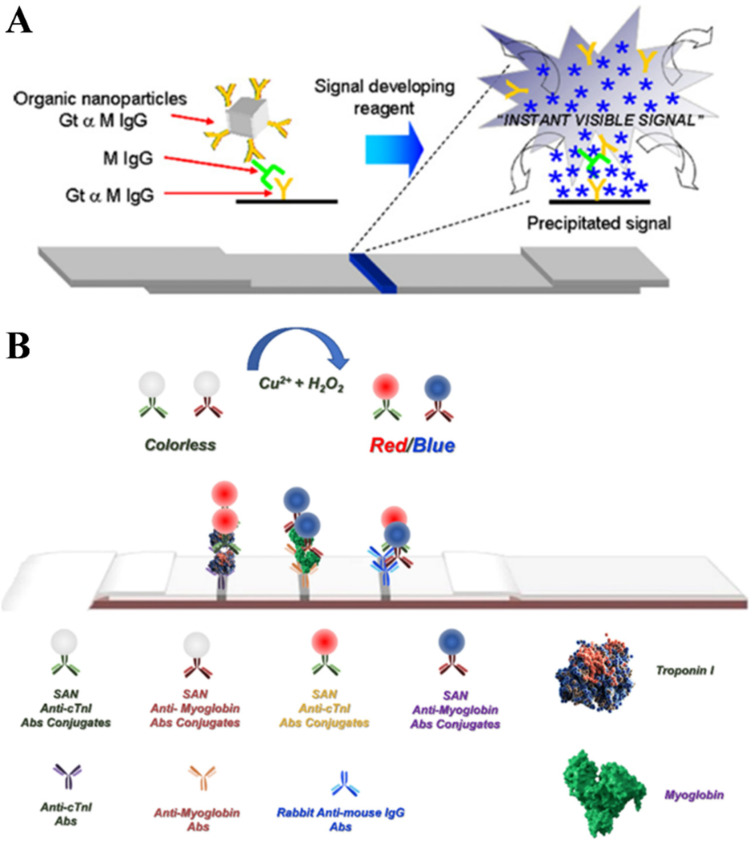
(**A**) Schematic diagram illustrating the principle of using organic nanoparticles as biolabels for immunodipsticks [119]. Copyright 2011 Elsevier. (**B**) Schematic representation of the strategy of integrating an SAN-LFA for the detection of cardiac biomarkers [120]. Copyright 2016 American Chemical Society.

Flow immunochromatographic assays are simple point-of-care testing devices that integrate the working principles of chromatography and immunochemical reactions. Compared with traditional ELISA, they have the advantages of high simplicity, rapid analysis, portability, and cost-effectiveness [118]. Self-assembled nanolabels have also been used for the design of flow immunochromatographic devices. For example, Mak et al. demonstrated that the 5-Bromo-4-chloro-3-indolyl acetate (BCIA) NPs could be used as the biolabels for immunodipsticks (Figure 10A) [119]. The BCIA NPs were prepared with BSA as the supernatant through a top-down method and then conjugated with EDC/NHS-activated antibody for target recognition. In the presence of analytes, the antibody-modified BCIA NPs were anchored on the test zone by sandwich immunoreactions. The colorless BCIA NPs were triggered by chemical induction to produce instant blue precipitates with the reagents of 2-propanol, NaOH and H_2_O_2_. In addition, Song et al. developed flow immunochromatographic assays using the self-assembling allochroic nanoparticles (SANs) of TMB and 3-amino-9-ethylcarbazole (AEC) monomers (Figure 10B) [120]. The TMB-based SANs and AEC-based SANs, denoted as TSANs and ASANs, respectively, were co-assembled with BSA and streptavidin for the attachment of biotinylated antibodies. Both TSANs and ASANs underwent oxidation in the presence of Cu^2+^/H_2_O_2_, exhibiting a visible color change in the test zone. Based on the change in blue/red color intensity, the contents of cardiac troponin I-troponin C (cTnI-TnC) and myoglobin (Myo) in plasma samples have been determined. The work inspires the self-assembled nanolabels to be integrated with flow immunochromatographic devices.

**Table 2:** Signaling molecules—assembled nanomaterials—as signal labels.

Signal Label	Target	Linear Range	Detection Limit	Ref.
FDA NCs	IgG	0–2 ng/mL	57 pg/mL	[103]
FDA NCs	IgG	4–100 ng/mL	4 ng/mL	[105]
FDA NCs	IgG	0–10 ng/mL	25.6 pg/mL	[107]
FDA NCs	CRP	0–100 ng/mL	1.1 ng/mL	[110]
Silole NCs	IgG	0–100 ng/mL	Not reported	[111]
TCPP NPs	IgG	10–1000 pM	2.05 pM	[112]
TMB NPs	IL-6	1–1000 pg/mL	0.66 pg/mL	[115]
TP-BE NPs, PP-BP NPs, CUR-BH NPs	ER, PR, and HER2	5–1000 pg/mL, 5–5000 pg/mL, 5–1000 pg/mL	1.92 pg/mL, 1.73 pg/mL, 2.64 pg/mL	[116]
BCIA NPs	IgG	1.25–20 ng/mL	1.25 ng/mL	[119]
TSANs, ASANs	cTnI-TnC and Myo	0.02–5 ng/mL, 0.5–5 ng/mL	0.012 ng/mL, 0.2 ng/mL	[120]

Abbreviation: FDA NCs, fluorescein diacetate nanocrystals; CRP, C-reactive protein; TCPP NPs, tetra(4-carboxyphenyl)porphyrin nanoparticles; TMB, 3,3′,5,5′-tetramethylbenzidine; IL-6, Interleukin-6; TP-BE NPs, thymolphthalein nanoparticles assembled with pH-BSA/ER antibody; PP-BP NPs, phenolphthalein nanoparticles assembled with pH-BSA/PR antibody; CUR-BH NPs, curcumin nanoparticles assembled with pH-BSA/PR antibody; ER, estrogen receptor; PR, progesterone receptor; HER2, human epidermal growth factor receptor-2; BCIA, 5-bromo-4-chloro-3-indolyl acetate; TSANs, TMB-based self-assembling allochroic nanocatalyst; ASANs, 3-amino-9-ethylcarbazole-based self-assembling allochroic nanocatalyst; cTnI-TnC, cardiac troponin I-troponin C; Myo, myoglobin.

## 4. Conclusions

Immunoassays have shown a wide range of application fields, such as clinical diagnosis, environmental monitoring, and food safety. Nanomaterials acting as enzyme mimics, signal indicators, and nanocarriers have been applied as the reporters of immunoassays with surprising performances. In this review, we summarized the preparation and application of signaling molecules-entrapped or assembled nanomaterials as signal labels for immunoassays. Such strategies improve the loading capacity of signaling molecules and the signal-to-noise ratio and obviate the use of natural enzymes. Despite exciting achievements of such methods for immunoassays of various analytes, there are still some issues remaining to be considered to meet the requirements of high sensitivity, simplicity, stability, and good repeatability of biosensors. First, active groups on the surface of nanocontainers are required to be functionalized with biological elements for molecular recognition, such as carboxy, hydroxy, sulfhydryl, or amino groups and unsaturated metal-binding sites. The complex modification procedures may limit the practical applications of the entrapped-nanolabel signaling molecules. Second, the fluorescent dye-based nanolabels do not perform well, usually due to the ACQ effect. For this, AIEgens can be designed as the signal labels of immunoassays due to their good self-assembly ability and unique optical properties. Third, the entrapped or assembled dye molecules are usually released by external stimuli to produce detectable signals for homogeneous analysis. Miniaturized devices can be integrated with immunoassays to determine the released signaling molecules in a small sample volume. Last but not least, most of the signal-on immunoassays refer to the use of a couple of antibodies, which will increase the analysis cost and require strict storage and detection conditions. Significant effort should be devoted to developing effective and stable artificial/synthetic receptors in place of the capture or recognition of antibodies in sandwich immunoassays, such as aptamers, lectins, boronic acids, and molecular imprinting materials. We believe that nanolabels prepared by the self-assembly of small molecules with multiple features, including molecular recognition and signal generation, may meet the requirement of sensitive, simple, and accurate immunoassays.

## Data Availability

Data are contained within the article.

## References

[1] Farka Z., Jurik T., Kovar D., Trnkova L., Skladal P. (2017). Nanoparticle-based immunochemical biosensors and assays: Recent advances and challenges. Chem. Rev..

[2] Pan R., Li G., Liu S., Zhang X., Liu J., Su Z., Wu Y. (2021). Emerging nanolabels-based immunoassays: Principle and applications in food safety. TrAC Trend. Anal. Chem..

[3] Farka Z., Brandmeier J.C., Mickert M.J., Pastucha M., Lacina K., Skladal P., Soukka T., Gorris H.H. (2024). Nanoparticle-based bioaffinity assays: From the research laboratory to the market. Adv. Mater..

[4] Kokkinos C., Economou A. (2017). Emerging trends in biosensing using stripping voltammetric detection of metal-containing nanolabels—A review. Anal. Chim. Acta.

[5] Svechkarev D., Mohs A.M. (2019). Organic fluorescent dye-based nanomaterials: Advances in the rational design for imaging and sensing applications. Curr. Med. Chem..

[6] Su D., Li H., Zhou R., Zhao L., Li A., Liu X., Wang C., Jia X., Liu F., Sun P. (2022). Embedding proteins within spatially controlled hierarchical nanoarchitectures for ultrasensitive immunoassay. Anal. Chem..

[7] Zhang Z., Xie J., Yu J., Lu Z., Liu Y. (2017). A novel colorimetric immunoassay strategy using iron(III) oxide magnetic nanoparticles as a label for signal generation and amplification. J. Mater. Chem. B.

[8] Zhang X., Li G., Wu D., Li X., Hu N., Chen J., Chen G., Wu Y. (2019). Recent progress in the design fabrication of metal-organic frameworks-based nanozymes and their applications to sensing and cancer therapy. Biosens. Bioelectron..

[9] Xia N., Liu G., Zhang S., Shang Z., Yang Y., Li Y., Liu L. (2022). Oxidase-mimicking peptide-copper complexes and their applications in sandwich affinity biosensors. Anal. Chim. Acta.

[10] Zhou L., Liu Y., Lu Y., Zhou P., Lu L., Lv H., Hai X. (2022). Recent advances in the immunoassays based on nanozymes. Biosensors.

[11] Guo J.-W., Yang Z.-W., Liu X.-L., Zhang L.-W., Guo W.-B., Zhang J., Ding L.-H. (2023). 2D Co metal-organic framework nanosheet as an oxidase-like nanozyme for sensitive biomolecule monitoring. Rare Met..

[12] Wang J., Wang X., Yang K., Hu S., Wang W. (2022). Self-assembly of small organic molecules into luminophores for cancer theranostic applications. Biosensors.

[13] Dai Z., Ju H. (2012). Bioanalysis based on nanoporous materials. TrAC Trend. Anal. Chem..

[14] Kumar P., Kim K.-H., Bansal V., Kumar S., Dilbaghi N., Kim Y.-H. (2017). Modern progress and future challenges in nanocarriers for probe applications. TrAC Trend. Anal. Chem..

[15] Hofmann C., Duerkop A., Baeumner A.J. (2019). Nanocontainers for analytical applications. Angew. Chem. Int. Ed..

[16] Climent E., Hecht M., Rurack K. (2021). Loading and release of charged and neutral fluorescent dyes into and from mesoporous materials: A key role for sensing applications. Micromachines.

[17] Wang Z., Guo Y., Xianyu Y. (2023). Applications of self-assembly strategies in immunoassays: A review. Coord. Chem. Rev..

[18] Patra A., Chandaluri Ch G., Radhakrishnan T.P. (2012). Optical materials based on molecular nanoparticles. Nanoscale.

[19] Liu Q., Boyd B.J. (2013). Liposomes in biosensors. Analyst.

[20] Almajidi Y.Q., Althomali R.H., Gandla K., Uinarni H., Sharma N., Hussien B.M., Alhassan M.S., Romero-Parra R.M., Bisht Y.S. (2023). Multifunctional immunosensors based on mesoporous silica nanomaterials as efficient sensing platforms in biomedical and food safety analysis: A review of current status and emerging applications. Microchem. J..

[21] Hu H., Wang Y. (2024). Recent advances in metal–organic frameworks as emerging platforms for immunoassays. TrAC Trend. Anal. Chem..

[22] Zhang X., Hu S., Huang L., Chen X., Wang X., Fu Y.-n., Sun H., Li G., Wang X. (2023). Advance progress in assembly mechanisms of carrier-free nanodrugs for cancer treatment. Molecules.

[23] Majdinasab M., de la Chapelle M.L., Marty J.L. (2023). Recent progresses in optical biosensors for Interleukin 6 detection. Biosensors.

[24] Mazur F., Bally M., Stadler B., Chandrawati R. (2017). Liposomes and lipid bilayers in biosensors. Adv. Colloid. Interface Sci..

[25] Siva S., Jin J.O., Choi I., Kim M. (2023). Nanoliposome based biosensors for probing mycotoxins and their applications for food: A review. Biosens. Bioelectron..

[26] Bacigalupo M.A., Ius A., Longhi R., Meroni G. (2003). Homogeneous immunoassay of atrazine in water by terbium-entrapping liposomes as fluorescent markers. Talanta.

[27] Bui M.P., Ahmed S., Abbas A. (2015). Single-digit pathogen and attomolar detection with the naked eye using liposome-amplified plasmonic immunoassay. Nano Lett..

[28] Edwards K.A., Baeumner A.J. (2007). DNA-oligonucleotide encapsulating liposomes as a secondary signal amplification means. Anal. Chem..

[29] Ganganboina A.B., Chowdhury A.D., Khoris I.M., Nasrin F., Takemura K., Hara T., Abe F., Suzuki T., Park E.Y. (2020). Dual modality sensor using liposome-based signal amplification technique for ultrasensitive norovirus detection. Biosens. Bioelectron..

[30] Orellana A., Laukkanen M.L., Keinanen K. (1996). Europium chelate-loaded liposomes: A tool for the study of binding and integrity of liposomes. Biochim. Biophys. Acta.

[31] Tran V.A., Doan V.D., Le V.T. (2024). Colorimetric sensor based on release control from liposomes by the formation of 2,2-bipyridine-Fe^2+^ complex. Microchem. J..

[32] Chang Y.F., Fu C., Chen Y.T., Fang-Ju Jou A., Chen C.C., Chou C., Annie Ho J.A. (2016). Use of liposomal amplifiers in total internal reflection fluorescence fiber-optic biosensors for protein detection. Biosens. Bioelectron..

[33] Ho J.A., Zeng S.C., Tseng W.H., Lin Y.J., Chen C.H. (2008). Liposome-based immunostrip for the rapid detection of Salmonella. Anal. Bioanal. Chem..

[34] Frost S.J., Chakraborty J., Firth G.B. (1996). Novel homogeneous liposomal immunoassay for colorimetric estimation of serum IgG anticardiolipin antibodies. Clin. Chem..

[35] Ho J.A., Wu L.C., Huang M.R., Lin Y.J., Baeumner A.J., Durst R.A. (2007). Application of ganglioside-sensitized liposomes in a flow injection immunoanalytical system for the determination of cholera toxin. Anal. Chem..

[36] Hofmann C., Kaiser B., Maerkl S., Duerkop A., Baeumner A.J. (2020). Cationic liposomes for generic signal amplification strategies in bioassays. Anal. Bioanal. Chem..

[37] Kondapalli S., Connelly J.T., Baeumner A.J., Kirby B.J. (2011). Integrated microfluidic preconcentrator and immunobiosensor. Microfluid. Nanofluidics.

[38] Liu X.Y., Nakamura C., Yang Q., Miyake J. (2001). Phospholipase A(2)-catalyzed membrane leakage studied by immobilized liposome chromatography with online fluorescent detection. Anal. Biochem..

[39] Wang B., Zhang L., Yin G., Wang J., Wang P., Wang T., Tian J., Yu X.-a., Chen H. (2022). Arg-liposome-amplified colorimetric immunoassay for selective and sensitive detection of cystatin C to predict acute kidney injury. Anal. Chim. Acta.

[40] Ren W., Ballou D.R., FitzGeraldc R., Irudayaraj J. (2019). Plasmonic enhancement in lateral flow sensors for improved sensing of E. coli O157:H7. Biosens. Bioelectron..

[41] Ren R., Cai G., Yu Z., Tang D. (2018). Glucose-loaded liposomes for amplified colorimetric immunoassay of streptomycin based on enzyme-induced iron(II) chelation reaction with phenanthroline. Sens. Actuators B Chem..

[42] Tang J., Huang Y., Liu H., Zhang C., Tang D. (2016). Novel glucometer-based immunosensing strategy suitable for complex systems with signal amplification using surfactant-responsive cargo release from glucose-encapsulated liposome nanocarriers. Biosens. Bioelectron..

[43] Mwanza D., Mfamela N., Adeniyi O., Nyokong T., Mashazi P. (2022). Ultrasensitive detection of prostate-specific antigen using glucose-encapsulated nanoliposomes anti-PSA polyclonal antibody as detection nanobioprobes. Talanta.

[44] Gu C., Gai P., Hou T., Li H., Xue C., Li F. (2017). Enzymatic fuel cell-based self-powered homogeneous immunosensing platform via target-induced glucose release: An appealing alternative strategy for turn-on melamine assay. CS Appl. Mater. Interfaces.

[45] Zhou S., Yuan L., Hua X., Xu L., Liu S. (2015). Signal amplification strategies for DNA and protein detection based on polymeric nanocomposites and polymerization: A review. Anal. Chim. Acta.

[46] Wu J., Xianyu Y., Wang X., Hu D., Zhao Z., Lu N., Xie M., Lei H., Chen Y. (2018). Enzyme-free amplification strategy for biosensing using Fe^3+^-poly(glutamic acid) coordination chemistry. Anal. Chem..

[47] Zhang Y., Ren F., Wang G., Liao T., Hao Y., Zhang H. (2021). Rapid and sensitive pathogen detection platform based on a lanthanide-labeled immunochromatographic strip test combined with immunomagnetic separation. Sens. Actuators B Chem..

[48] Yang Y., Su Z., Wu D., Liu J., Zhang X., Wu Y., Li G. (2022). Low background interference SERS aptasensor for highly sensitive multiplex mycotoxin detection based on polystyrene microspheres-mediated controlled release of Raman reporters. Anal. Chim. Acta.

[49] Zhu H., McShane M.J. (2005). Loading of hydrophobic materials into polymer particles: Implications for fluorescent nanosensors and drug delivery. J. Am. Chem. Soc..

[50] Behnke T., Wurth C., Hoffmann K., Hubner M., Panne U., Resch-Genger U. (2011). Encapsulation of hydrophobic dyes in polystyrene micro- and nanoparticles via swelling procedures. J. Fluoresc..

[51] Geißler D., Wegner K.D., Fischer C., Resch-Genger U. (2024). Exploring simple particle-based signal amplification strategies in a heterogeneous sandwich immunoassay with optical detection. Anal. Chem..

[52] Sancenon F., Pascual L., Oroval M., Aznar E., Martinez-Manez R. (2015). Gated silica mesoporous materials in sensing applications. ChemistryOpen.

[53] Coll C., Bernardos A., Martinez-Manez R., Sancenon F. (2013). Gated silica mesoporous supports for controlled release and signaling applications. Acc. Chem. Res..

[54] Behyar M.B., Nilghaz A., Hasanzadeh M., Shadjou N. (2024). Recent progresses and challenges on mesoporous silica nanoparticles for DNA-based biosensors and diagnostics. TrAC Trend. Anal. Chem..

[55] Wei W., Wei M., Liu S. (2012). Silica nanoparticles as a carrier for signal amplification. Rev. Anal. Chem..

[56] Mladenović M., Jarić S., Mundžić M., Pavlović A., Bobrinetskiy I., Knežević N.Ž. (2024). Biosensors for cancer biomarkers based on mesoporous silica nanoparticles. Biosensors.

[57] Parra M., Gil S., Gavina P., Costero A.M. (2021). Mesoporous silica nanoparticles in chemical detection: From small species to large bio-molecules. Sensors.

[58] Gai P., Gu C., Li H., Sun X., Li F. (2017). Ultrasensitive ratiometric homogeneous electrochemical microRNA biosensing via target-triggered Ru(III) release and redox recycling. Anal. Chem..

[59] Ma H., Wang Y., Wu D., Zhang Y., Gao J., Ren X., Du B., Wei Q. (2016). A novel controlled release immunosensor based on benzimidazole functionalized SiO_2_ and cyclodextrin functionalized gold. Sci. Rep..

[60] Shams A., Zarif B.R., Salouti M., Shapouri R., Mirzaii S. (2019). Designing an immunosensor for detection of Brucella abortus based on coloured silica nanoparticles. Artif. Cells Nanomed. Biotechnol..

[61] Wang P., Wang B., Chen Y., Lin N., Zheng Z., Chen H., Wang W., He Y. (2024). Highly selective detection of breast cancer cells mediated by multi-aptamer and dye-loaded mesoporous silica nanoparticles. Microchim. Acta.

[62] Sun Q., Zhao G., Dou W. (2015). Blue silica nanoparticle-based colorimetric immunoassay for detection of Salmonella pullorum. Anal. Methods.

[63] Shao F., Zhang L., Jiao L., Wang X., Miao L., Li H., Zhou F. (2018). Enzyme-free immunosorbent assay of prostate specific antigen amplified by releasing pH indicator molecules entrapped in mesoporous silica nanoparticles. Anal. Chem..

[64] Xia N., Deng D., Mu X., Liu A., Xie J., Zhou D., Yang P., Xing Y., Liu L. (2020). Colorimetric immunoassays based on pyrroloquinoline quinone-catalyzed generation of Fe(II)-ferrozine with tris(2-carboxyethyl)phosphine as the reducing reagent. Sens. Actuators B Chem..

[65] Tang D., Liu B., Niessner R., Li P., Knopp D. (2013). Target-induced displacement reaction accompanying cargo release from magnetic mesoporous silica nanocontainers for fluorescence immunoassay. Anal. Chem..

[66] Hu R.R., Yin Z.Z., Zeng Y.B., Zhang J., Liu H.Q., Shao Y., Ren S.B., Li L. (2016). A novel biosensor for Escherichia coli O157:H7 based on fluorescein-releasable biolabels. Biosens. Bioelectron..

[67] Ghafary Z., Hallaj R., Salimi A., Mafakheri S. (2021). Ultrasensitive fluorescence immunosensor based on mesoporous silica and magnetic nanoparticles: Capture and release strategy. Spectrochim. Acta Part A Mol. Biomol. Spectrosc..

[68] Hecht M., Climent E., Biyikal M., Sancenón F., Martínez-Máñez R., Rurack K. (2013). Gated hybrid delivery systems: En route to sensory materials with inherent signal amplification. Coord. Chem. Rev..

[69] Climent E., Bernardos A., Martinez-Manez R., Maquieira A., Marcos M.D., Pastor-Navarro N., Puchades R., Sancenon F., Soto J., Amoros P. (2009). Controlled delivery systems using antibody-capped mesoporous nanocontainers. J. Am. Chem. Soc..

[70] Costa E., Climent E., Ast S., Weller M.G., Canning J., Rurack K. (2020). Development of a lateral flow test for rapid pyrethroid detection using antibody-gated indicator-releasing hybrid materials. Analyst.

[71] Costa E., Climent E., Gawlitza K., Wan W., Weller M.G., Rurack K. (2020). Optimization of analytical assay performance of antibody-gated indicator-releasing mesoporous silica particles. J. Mater. Chem. B.

[72] Climent E., Biyikal M., Gröninger D., Weller M.G., Martínez-Máñez R., Rurack K. (2020). Multiplex-nachweis von analyten auf einem einzelnen teststreifen mit antikörper-gesteuerten und indikator freisetzenden mesoporösen nanopartikeln. Angew. Chem..

[73] Climent E., Groninger D., Hecht M., Walter M.A., Martinez-Manez R., Weller M.G., Sancenon F., Amoros P., Rurack K. (2013). Selective, sensitive, and rapid analysis with lateral-flow assays based on antibody-gated dye-delivery systems: The example of triacetone triperoxide. Chem. Eur. J..

[74] Pla L., Lozano-Torres B., Martinez-Manez R., Sancenon F., Ros-Lis J.V. (2019). Overview of the evolution of silica-based chromo-fluorogenic nanosensors. Sensors.

[75] Climent E., Weller M.G., Martínez-Máñez R., Rurack K. (2021). Immunochemical design of antibody-gated indicator delivery (gaid) systems based on mesoporous silica nanoparticles. ACS Appl. Nano Mater..

[76] Zhu N., Zou Y., Huang M., Dong S., Wu X., Liang G., Han Z., Zhang Z. (2018). A sensitive, colorimetric immunosensor based on Cu-MOFs and HRP for detection of dibutyl phthalate in environmental and food samples. Talanta.

[77] Hu X., Wei Z., Sun C., Long Y., Zheng H. (2020). Bifunctional antibody and copper-based metal-organic framework nanocomposites for colorimetric alpha-fetoprotein sensing. Microchim. Acta.

[78] Yan H., Jiao L., Wang H., Xu W., Wu Y., Gu W., Du D., Lin Y., Zhu C. (2019). A “sense-and-treat” ELISA using zeolitic imidazolate framework-8 as carriers for dual-modal detection of carcinoembryonic antigen. Sens. Actuators B Chem..

[79] Chen Y., Mo J., Chen D., Chen P., Yang L. (2024). Colorimetric detection of Fe^2+^ and Cr_2_O_7_^2−^ in environmental water samples based on dual-emitting RhB-embedded Zr-MOFs. Spectrochim. Acta Part A Mol. Biomol. Spectrosc..

[80] Li X., Gao X., Gai P., Liu X., Li F. (2020). Degradable metal-organic framework/methylene blue composites-based homogeneous electrochemical strategy for pesticide assay. Sens. Actuators B Chem..

[81] Wei J., Zhang D., Zhang L., Ouyang H., Fu Z. (2019). Alkaline hydrolysis behavior of metal-organic frameworks NH_2_-MIL-53(Al) employed for sensitive immunoassay via releasing fluorescent molecules. ACS Appl. Mater. Interfaces.

[82] Wei D., Xiong D., Zhu N., Wang Y., Hu X., Zhao B., Zhou J., Yin D., Zhang Z. (2022). Copper peroxide nanodots encapsulated in a metal−organic framework for self-supplying hydrogen peroxideand signal amplification of the dual-mode immunoassay. Anal. Chem..

[83] Wu S., Li C., Shi H., Huang Y., Li G. (2018). Design of metal-organic framework-based nanoprobes for multicolor detection of DNA targets with improved sensitivity. Anal. Chem..

[84] Li C., Feng X., Yang S., Xu H., Yin X., Yu Y. (2021). Capture, detection, and simultaneous identification of rare circulating tumor cells based on a rhodamine 6G-loaded metal-organic framework. ACS Appl. Mater. Interfaces.

[85] Wang Y., Chen L., Wu Q., Wen Z., Ren Y., Wang M. (2019). An acid-responsive all-in-one signal amplification strategy for the ultrasensitive prostate-specific antigen detection. New J. Chem..

[86] Shee N.K., Kim H.-J. (2024). Porphyrin-based nanomaterials for the photocatalytic remediation of wastewater: Recent advances and perspectives. Molecules.

[87] Chen J., Zhu Y., Kaskel S. (2021). Porphyrin-based metal–organic frameworks for biomedical applications. Angew. Chem. Int. Ed..

[88] Wei Y.-J., Li J., Hu Z.-E., Xing X., Zhou Z.-W., Yu Y., Yu X.-Q., Zhang J., Liu Y.-H., Wang N. (2023). A porphyrin-MOF-based integrated nanozyme system for catalytic cascades and light-enhanced synergistic amplification of cellular oxidative stress. J. Mater. Chem. B.

[89] Duan H., Li D., Wang J., Shen Y., Zheng L., Huang X. (2024). A cocatalytic nanozyme based on metal-organic framework-embedded iron porphyrin for the sensitive detection of Salmonella typhimurium in milk. Talanta.

[90] Gutierrez M., Zhang Y., Tan J.C. (2022). Confinement of luminescent guests in metal-organic frameworks: Understanding pathways from synthesis and multimodal characterization to potential applications of LG@MOF systems. Chem. Rev..

[91] d’Ischia M., Napolitano A., Ball V., Chen C.-T., Buehler M.J. (2014). Polydopamine and eumelanin: From structure-property relationships to a unified tailoring strategy. Acc. Chem. Res..

[92] Ren R., Cai G., Yu Z., Zeng Y., Tang D. (2018). Metal-polydopamine framework: An innovative signal-generation tag for colorimetric immunoassay. Anal. Chem..

[93] Gao F., Chang Y., Zhang J., Wang L., Liu L. (2023). Stimuli-responsive aggregation-induced emission of molecular probes by electrostatic and hydrophobic interactions: Effect of organic solvent content and application for probing of alkaline phosphatase activity. Talanta.

[94] Gao F., Liu G., Qiao M., Li Y., Yi X. (2022). Biosensors for the detection of enzymes based on aggregation-induced emission. Biosensors.

[95] Liu G., Gao F., Yang X., Zhang J., Yang S., Li Y., Liu L. (2023). Aggregation-induced emission for the detection of peptide ligases with improving ligation efficiency. Anal. Chim. Acta.

[96] Wu W., Li Y., Song P., Xu Q., Lei D., Wang J., Fu B., Kong W. (2024). UiOL@AIEgens-assisted lateral flow immunosensor for the ultrasensitive dual-modal point-of-care detection of aflatoxin B(1). J. Hazard. Mater..

[97] Pu Q., Yang X., Guo Y., Dai T., Yang T., Ou X., Li J., Sheng S., Xie G. (2019). Simultaneous colorimetric determination of acute myocardial infarction biomarkers by integrating self-assembled 3D gold nanovesicles into a multiple immunosorbent assay. Microchim. Acta.

[98] Ghisaidoobe A.B., Chung S.J. (2015). Functionalized protein nanocages as a platform of targeted therapy and immunodetection. Nanomedicine.

[99] Song N., Zhang J., Zhai J., Hong J., Yuan C., Liang M. (2021). Ferritin: A multifunctional nanoplatform for biological detection, imaging diagnosis, and drug delivery. Acc. Chem. Res..

[100] Shao F., Jiao L., Miao L., Wei Q., Li H. (2017). A pH Indicator-linked Immunosorbent assay following direct amplification strategy for colorimetric detection of protein biomarkers. Biosens. Bioelectron..

[101] Zhou Y., Huang X., Xiong S., Li X., Zhan S., Zeng L., Xiong Y. (2018). Dual-mode fluorescent and colorimetric immunoassay for the ultrasensitive detection of alpha-fetoprotein in serum samples. Anal. Chim. Acta.

[102] Bu T., Zhao S., Bai F., Sun X., He K., Wang Q., Jia P., Tian Y., Zhang M., Wang L. (2021). Diverse dyes-embedded Staphylococcus aureus as potential biocarriers for enhancing sensitivity in biosensing. Anal. Chem..

[103] Chan C.P., Bruemmel Y., Seydack M., Sin K.K., Wong L.W., Merisko-Liversidge E., Trau D., Renneberg R. (2004). Nanocrystal biolabels with releasable fluorophores for immunoassays. Anal. Chem..

[104] Fery-Forgues S. (2013). Fluorescent organic nanocrystals and non-doped nanoparticles for biological applications. Nanoscale.

[105] Trau D., Yang W., Seydack M., Caruso F., Yu N.T., Renneberg R. (2002). Nanoencapsulated microcrystalline particles for superamplified biochemical assays. Anal. Chem..

[106] Chan C.P.-y., Tzang L.C.-h., Sin K.-k., Ji S.-l., Cheung K.-y., Tam T.-k., Yang M.M.-s., Renneberg R., Seydack M. (2007). Biofunctional organic nanocrystals for quantitative detection of pathogen deoxyribonucleic acid. Anal. Chim. Acta.

[107] Sin K.K., Chan C.P., Pang T.H., Seydack M., Renneberg R. (2006). A highly sensitive fluorescent immunoassay based on avidin-labeled nanocrystals. Anal. Bioanal. Chem..

[108] Bruemmel Y., Chan C.P., Renneberg R., Thuenemann A., Seydack M. (2004). On the influence of different surfaces in nano- and submicrometer particle based fluorescence immunoassays. Langmuir.

[109] Kamyshny A., Magdassi S. (2000). Fluorescence immunoassay based on fluorescer microparticles. Colloids Surf. B.

[110] Sin K.K., Chan C.P., Leung W.M., Seydack M., Renneberg R. (2006). Fluorogenic nanocrystals for highly sensitive detection of C-reactive protein. IEE Proc. Nanobiotechnol..

[111] Chan C.P., Haeussler M., Zhong Tang B., Dong Y., Sin K.K., Mak W.C., Trau D., Seydack M., Renneberg R. (2004). Silole nanocrystals as novel biolabels. J. Immunol. Methods.

[112] Gibson L.E., Wright D.W. (2016). Sensitive method for biomolecule detection utilizing signal amplification with porphyrin nanoparticles. Anal. Chem..

[113] Sun X., Li Y., Yang Q., Xiao Y., Zeng Y., Gong J., Wang Z., Tan X., Li H. (2021). Self-assembled all-inclusive organic-inorganic nanoparticles enable cascade reaction for the detection of glucose. Chin. Chem. Lett..

[114] Khoris I.M., Ganganboina A.B., Suzuki T., Park E.Y. (2021). Self-assembled chromogen-loaded polymeric cocoon for respiratory virus detection. Nanoscale.

[115] Jiao L., Yan H., Xu W., Wu Y., Gu W., Li H., Du D., Lin Y., Zhu C. (2019). Self-assembly of all-inclusive allochroic nanoparticles for the improved ELISA. Anal. Chem..

[116] Xu W., Jiao L., Ye H., Guo Z., Wu Y., Yan H., Gu W., Du D., Lin Y., Zhu C. (2020). pH-responsive allochroic nanoparticles for the multicolor detection of breast cancer biomarkers. Biosens. Bioelectron..

[117] Yan C., Sun Y., Yao M., Jin X., Yang Q., Wu W. (2022). pH-responsive nanoparticles and automated detection apparatus for dual detection of pathogenic bacteria. Sens. Actuators B Chem..

[118] Ma X., Ge Y., Xia N. (2024). Overview of the design and application of dual-signal immunoassays. Molecules.

[119] Mak W.C., Sin K.K., Chan C.P., Wong L.W., Renneberg R. (2011). Biofunctionalized indigo-nanoparticles as biolabels for the generation of precipitated visible signal in immunodipsticks. Biosens. Bioelectron..

[120] Song Y., Cai X., Ostermeyer G., Yu J., Du D., Lin Y. (2021). Self-assembling allochroic nanocatalyst for improving nanozyme-based immunochromatographic assays. ACS Sens..

